# The Need for Individualized Risk Assessment in Cardiovascular Disease

**DOI:** 10.3390/jpm12071140

**Published:** 2022-07-14

**Authors:** Hui Yin Lim, Louise M. Burrell, Rowena Brook, Harshal H. Nandurkar, Geoffrey Donnan, Prahlad Ho

**Affiliations:** 1 Northern Pathology Victoria, Northern Health, Epping, Melbourne, VIC 3076, Australia; huiyin.lim@nh.org.au (H.Y.L.); rowena.brook@nh.org.au (R.B.); 2 Department of Hematology, Northern Health, Epping, Melbourne, VIC 3076, Australia; 3 Australian Centre for Blood Diseases, Monash University, Melbourne, VIC 3004, Australia; harshal.nandurkar@monash.edu; 4 Department of Medicine, Northern Health, University of Melbourne, Epping, Melbourne, VIC 3076, Australia; 5 Department of Medicine, Austin Health, University of Melbourne, Heidelberg, Melbourne, VIC 3084, Australia; l.burrell@unimelb.edu.au; 6 The Melbourne Brain Centre, Royal Melbourne Hospital, University of Melbourne, Parkville, Melbourne, VIC 3010, Australia; geoffrey.donnan@unimelb.edu.au

**Keywords:** cardiovascular disease, risk assessment, Virchow’s triad, global coagulation assays, endothelial markers

## Abstract

Cardiovascular disease remains the leading cause of death in the era of modern medicine despite major advancements in this field. Current available clinical surrogate markers and blood tests do not adequately predict individual risk of cardiovascular disease. A more precise and sophisticated tool that can reliably predict the thrombosis and bleeding risks at an individual level is required in order for clinicians to confidently recommend early interventions with a favorable risk–benefit profile. Critical to the development of this tool is the assessment and understanding of Virchow’s triad and its complex interactions between hypercoagulability, endothelial dysfunction and vessel flow, a fundamental concept to the development of thrombosis. This review explores the pathophysiology of cardiovascular disease stemming from the triad of factors and how individualized risk assessment can be improved through the multimodal use of tools such as global coagulation assays, endothelial biomarkers and vessel flow assessment.

## 1. Introduction

Cardiovascular disease is a major global cause of death, with an estimated 17.9 million cardiovascular disease-related deaths in 2019, representing nearly one third of global deaths [1]. The Framingham Heart Study reports the lifetime risk of coronary heart disease, which accounts for up to half of the total cases of cardiovascular disease at age 40 in 49% of men and 32% of women [2]. The majority of cardiovascular risk factors are known and potentially modifiable; risk factors such as smoking, dyslipidemia, hypertension, diabetes and abdominal obesity account for more than half of cardiovascular mortality [3]. Despite the significant achievements in cardiovascular medicine, the burden of risk factors remains substantial with the increasing prevalence of an aging population, obesity, hypertension and diabetes. Moreover, there can be a wide range of cardiovascular outcomes in different individuals in response to the same risk factors, despite appropriate pharmacological and lifestyle optimization. A small proportion of individuals may manifest with cardiovascular disease despite no known prior risk factors. These observations highlight the knowledge gap in risk stratifying patients.

Despite the significant improvements in clinical risk assessment and the availability of effective clinical interventions, the burden of cardiovascular disease remains unacceptably high, and better risk assessment tools to predict future cardiovascular events are required. The lack of precision of the clinical surrogate markers and coagulation tests to predict individual cardiovascular risk underscores the need for better risk assessment tools. Critical to furthering our understanding of cardiovascular and thrombotic risk is the recognition that the pathophysiology is often multifactorial and requires a detailed understanding of Virchow’s triad. The complex interactions between all three elements, hypercoagulability, endothelial dysfunction and flow stasis, are pivotal to the development of thrombosis [4]. This paper aims to explore the pathophysiology of cardiovascular disease stemming from the triad of factors, the limitations of current risk assessment models and how individualized risk assessment can be improved through the multimodal use of tools such as global coagulation assays, endothelial biomarkers and vessel flow assessment.

## 2. Traditional Risk Factors for Cardiovascular Disease

Atherosclerosis, which can begin in childhood, is a multifactorial, chronic condition that contributes to cardiovascular disease. Characterized by lipid deposition in the blood vessel intima, atherosclerosis is associated with inflammation and calcification and can cause vessel stenosis with thrombotic occlusion and/or embolism [5]. The risk factors for atherosclerosis include diabetes mellitus, smoking, male gender, older age, obesity, hyperlipidemia, genetic abnormalities, hypertension and chronic inflammation [5,6,7]. While the modifiable risk factors such as diabetes, obesity and hyperlipidemia, have evolved over time with societal awareness and lifestyles, there remains significant heterogeneity in the clinical manifestations of these risk factors and associated cardiovascular complications.

Advancing age confers a major risk for cardiovascular diseases including coronary disease, hypertension, congestive heart failure and stroke. There are several mechanisms for the dominant effect of age including increased exposure time to age-dependent risk factors that may co-vary in number and severity with aging as well as changes in cardiovascular structure and function with age and how these changes interact with pathophysiological disease mechanisms [8]. Aging, as described by Lakatta, is a manifestation of progressive, time-dependent failure molecular disorder that compromises cardiovascular reserve function [9]. In an attempt to limit the molecular disorder, the cardiovascular cells generate an inflammatory defense, through the activation of renin–angiotensin–aldosterone endothelin signaling cascades. Advancing age is also associated with the increased prevalence of salt-sensitive hypertension, which is also linked to central arterial stiffness. The damaged arterial wall due to pulsatile injury contributes to the proinflammatory state [10]. Over time, the age-associated inflammation in the heart and arteries exceeds a threshold and together with aging-related hemodynamic alterations contributes to fibrosis and arterial stiffness, resulting in cardiovascular diseases [9].

Diabetes patients are predisposed to premature atherosclerosis and increased cardiovascular disease burden. Hyperglycemia and insulin resistance have been associated with platelet hyper-reactivity and increased procoagulant factors such as tissue factor and factor VII, in addition to hypofibrinolysis [11,12,13]. In addition to slower clot lysis, the clot structure in diabetes has also previously been shown to have a more compact fibrin network [13]. Hyperglycemia further impairs endothelial function by inducing inflammation and oxidative stress, further promoting platelet aggregation. Moreover, diabetes patients are also predisposed to plaque rupture and thrombus formation.

Patients with known cardiovascular risk factors often have one or more other risk factors. For instance, up to 97% of diabetic patients are reported to have concurrent dyslipidemia. Furthermore, the predominant form of low-density lipoprotein (LDL) in diabetes is the more atherogenic small LDL particles with hyperinsulinemia, which further promote the dysregulation of lipid metabolism, resulting in hypertriglyceridemia, both critical to the process of atherosclerosis [14]. Cholesterol accumulates in foam cells (lipid-laden macrophages), leading to mitochondrial dysfunction and apoptosis, with the resultant release in inflammatory cytokines and prothrombotic molecules [15].

Another highly prevalent condition is obesity, which is now rapidly becoming a global health pandemic. It is estimated that the prevalence of obesity has nearly tripled since 1975, with 52% of adults being either overweight or obese [16]. Importantly, the Framingham Offspring Study reported that obesity independently predicted cardiovascular risk factors after adjustment for traditional risks [17]. Both visceral and central obesity promote systemic and vascular inflammation, which is fundamental to the development and acceleration of atherosclerotic changes [18]. Obesity also tends to co-exist with other risk factors for atherosclerosis including insulin resistance, hypertension and dyslipidemia, as well as playing an important role in perpetuating these atherogenic pathways. The Metabolic syndrome and Artery Research (MARE) Consortium also reported that specific clusters of metabolic syndromes (high triglycerides–high blood pressure–abdominal obesity, low high-density-lipoprotein (HDL) cholesterol–high blood pressure–abdominal obesity, high glucose–high blood pressure–abdominal obesity) were associated with a 50–90% greater likelihood of presenting with extremely thick (older) large (carotid) arteries [19].

## 3. Current Available Risk Assessment Tools

The current available risk assessment models are predominantly aimed at estimating the risk of primary cardiovascular events in asymptomatic individuals over a relatively short time frame of 5 to 10 years, a significant limitation of these models [20]. Largely based on traditional risk factors, these models often do not adequately consider nontraditional risk factors such as metabolic syndrome, chronic inflammation, gestational syndromes and other determinants of health, and they also differ in the included variables and evaluated endpoints. Few of these models adopt the use of biomarkers despite the growing evidence of the usefulness of circulating and imaging biomarkers. Table 1 shows the components of various widely used risk assessment models [21,22,23,24,25].

The applicability of the absolute risk calculators is often not generalizable to every individual or population, and there is no single risk model that is appropriate for all patients. The performance of a risk calculator is also dependent on the population characteristics and availability of primary preventive therapies. DeFilippis et al. compared several risk scores in the same population and found significant variability in the predicted risk [26]. Furthermore, current guidelines stratify the risks between high or low risk groups, and the intermediate group lacks clarity for guiding clinicians on how to approach the primary prevention in these patients. In addition, the calculators are not suitable for those with overt cardiovascular disease as by definition, these are already high-risk patients. However, it would be ideal to be able to pinpoint individuals, including those deemed high-risk, who are at impending risk of cardiovascular events to enable clinicians to recommend timely interventions.

## 4. Virchow’s Triad and Its Association with Thrombosis and Cardiovascular Disease

Crucial to the development of thrombosis is the concept of Virchow’s triad. The complex interplay between three elements—the presence of hypercoagulable state, flow stasis and endothelial injury or dysfunction—provides the perfect storm for thrombogenesis [4]. Traditionally used to describe the pathophysiology of venous thrombosis, it has become evident that these three factors are also pertinent to the evolution of arterial thrombosis [4,27,28] and hence cardiovascular disease. In addition, atherosclerosis has been closely linked to venous thromboembolism (VTE); patients with VTE appear to have common risk factors with atherosclerosis, and patients with unprovoked VTE have also demonstrated higher prevalences of arterial thrombosis compared with healthy controls [29,30].

**Figure 1:**
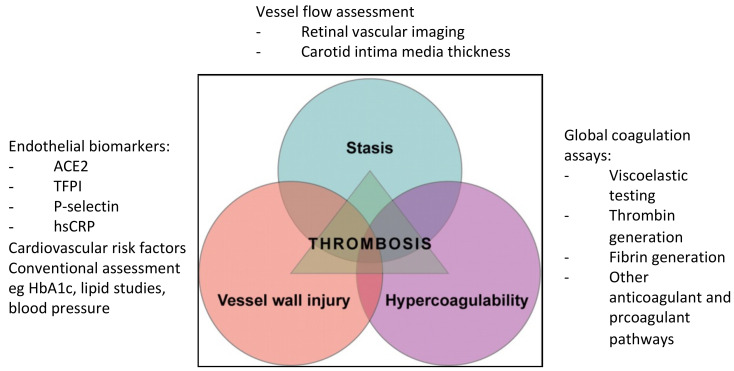
Some of the key biomarkers used in the assessment of the various components of Virchow’s triad. (Abbreviations: ACE2 angiotensin converting enzyme 2; TFPI tissue factor pathway inhibitor; hsCRP high-sensitivity C-reactive protein; HbA1c glycated hemoglobin).

In atherosclerosis, the triad are representative of abnormalities in the endothelium, platelets and coagulation and fibrinolytic pathways, as well as in hemorheology and turbulence at bifurcation and stenotic regions [4,27,28]. Atherosclerosis is a result of chronic inflammation involving circulating factors, endothelial cells, vascular smooth muscle cells and mononuclear cells due to repeated exposure to vascular injury such as plasma lipids, particularly low-density lipoproteins. It has been postulated that platelets play a major role in atherogenesis through adhesion to the endothelium as a result of vascular wall injury and leucocyte recruitment in atherosclerotic plaque [31]. Plaque rupture exposes prothrombotic proteins including collagen and tissue factor to the coagulation system resulting in thrombus formation, restricted blood flow and onset of cardiovascular disease. The activated platelets also interact with the atherosclerotic endothelium, resulting in the delivery of pro-inflammatory chemokines, further propagating the inflammatory process. Figure 1 shows some key biomarkers that can be used to assess the components of Virchow’s triad.

### 4.1. Assessment of Hypercoagulability with Global Coagulation Assays

**Figure 2:**
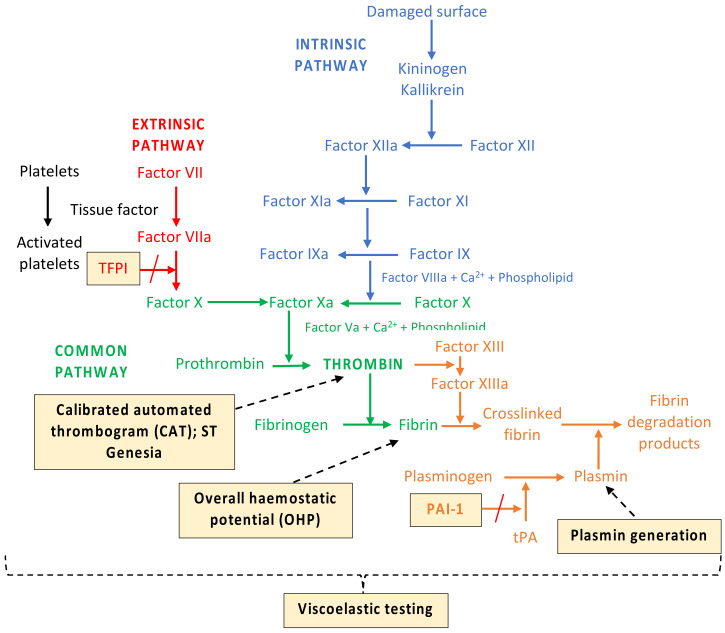
A simplified coagulation cascade highlighting the key pathways examined by some of the available global coagulation assays (highlighted boxes) (Abbreviations: TFPI tissue factor pathway inhibitor; PAI-1 plasminogen activator inhibitor-1; tPA tissue plasminogen activator).

Coagulation also plays an important role in atherogenesis. It is a dynamic but tightly regulated process. The exposure of tissue factor to blood through damage to the endothelium represents the initiation phase of the coagulation, resulting in a stepwise activation of coagulation zymogens [32]. This process leads to the amplification of the clotting factors and accelerated thrombin production, which further enhances platelet aggregation and the activation of the intrinsic pathway enzymes. Thrombin is an important enzyme that regulates the procoagulant–anticoagulant balance as well as several cellular processes through protease–activator receptors (PARs). Local coagulation activity and the end-product fibrin are a result of arterial vessel wall damage inflicted by atherogenesis [33]. Patients with diabetes, for instance, demonstrate platelet hyperactivity with elevated coagulation activation markers such as prothrombin fragment 1+2 and thrombin–antithrombin complexes as well as clotting factors including fibrinogen; factors VII, VIII, XI and XII; and von Willebrand factor [12]. While conventional coagulation assays are not useful in predicting thrombosis risks, global coagulation assays provide a more comprehensive assessment of coagulation including the evaluation of the final products of the coagulation cascade, thrombin and fibrin [34,35]. Although limited in their clinical use in thrombosis medicine in the current state, these assays appear promising, particularly in detecting hypercoagulable states [27,35]. Figure 2 shows some of the available global coagulation assays.

Viscoelastic testing such as thromboelastography and rotational thromboelastometry measures the changes in the elastic properties of whole blood during clot formation and propagation, clot firmness and fibrinolytic dissolution [36]. It is clinically used to guide transfusion requirements in bleeding and trauma situations [35], and it is reported to be more hypercoagulable in diabetes, stroke, hematological and oncological malignancies, and pregnancy [27,37,38,39] in small observational studies. Furthermore, Rafiq et al. found that patients undergoing coronary artery bypass grafting surgery with hypercoagulable maximum amplitude (a measure of clot strength) on thromboelastography had a higher risk of composite thromboembolic complications and mortality after surgery [40].

Similar to viscoelastic testing, thrombin generation assays such as calibrated automated thrombogram (CAT) are superior to conventional coagulation testing in detecting hypercoagulability, although their role in predicting future cardiovascular events remains unanswered [34,35]. While a large study of over 9000 subjects aged >65 years old demonstrated that thrombin generation positively correlated with the risk of acute ischemic stroke in women [41], the LURIC study found that individuals undergoing coronary angiography with the lowest endogenous thrombin potential quartile (amount of thrombin) had lower event-free survival [42]. Interestingly, we have reported that healthy controls with “flattened” thrombin generation curves (those with lower velocity index (rate of thrombin generation) despite preserved endogenous thrombin potential on CAT)) paradoxically demonstrated poorer lipid profiles and increased P-selectin and tissue factor pathway inhibitor (TFPI) [43]. This suggests that there are complex interactions in the coagulation system that warrant further investigation.

Thrombin activation can also lead to fibrin formation through the crosslinking of factor XIIIa fibrin monomers, although this is counterbalanced by anticoagulant mechanisms including TFPI and thrombin itself. In addition to triggering the coagulation cascade, vascular endothelial injury also activates the fibrinolytic system. This balance is maintained by the interplay between tissue-type plasminogen activator (t-PA), promoter of fibrinolysis, and plasminogen activator inhibitor-1 (PAI-1), inhibitor of fibrinolysis. The fibrin clot is removed by the plasmin, released from endothelial cells in response to thrombin, although clot structure in diabetes has previously been shown to have more “compact” fibrin network structure with reduced permeability [13]. Impaired fibrinolysis has also been demonstrated in patients with diabetes and those with cardiovascular disease [4]. A study demonstrated that glycated hemoglobin levels (HbA1c) positively correlated with PAI-1 activity (*r* = 0.50, *p* = 0.002) and negatively correlated with tPA activity (*r* = −0.452, *p* = 0.004) [44]. Previous studies using the overall hemostatic potential assay (OHP), a spectrophotometric assessment of fibrin aggregation, have shown that the intake of omega-3 polyunsaturated can reduce fibrin generation in healthy subjects [45] and that the overall fibrinolytic potential is impaired in patients with diabetes and those with stable coronary heart disease [37,46]. Another more recent assay to show potential in identifying fibrinolytic dysfunction is plasmin generation using a modified CAT technique by Miszta et al. [47] that was delayed in the plasma of mice fed a high-fat diet to induce obesity [48].

### 4.2. Endothelial Dysfunction

The endothelium is highly plastic and diverse and is very amenable in both health and disease. The vascular endothelial cells play critical roles in regulating the crosstalk of the various components required for the maintenance of cardiovascular homeostasis. These components include the regulation of blood constituents, vascular tone, angiogenesis, leucocyte adhesion and platelet aggregation [49,50]. Located between tissue and blood, endothelial cells play an integral role in protection against vascular injury and the maintenance of blood fluidity [28]. Endothelial dysfunction is associated with increased vascular permeability and the decreased production of nitric oxide as well as increased reactive oxygen species (ROS) and proinflammatory factors, and it is induced by oxidized LDL [5,51]. The activated pro-atherogenic endothelium increases lipid uptake into the vessel wall and expresses surface adhesion molecules, which bind circulating inflammatory cells and secrete cytokines and chemokines to further attract the inflammatory cells as well as secrete growth factors which promote vascular smooth muscle cell proliferation and intimal hyperplasia [5,51]. Several chronic metabolic conditions such as the previously listed traditional risk factors of cardiovascular disease can lead to a loss of this equilibrium, shifting towards endothelial dysfunction, a key pathophysiology in atherosclerosis.

There are several ways to measure endothelial function. Kitta et al. utilized the flow-mediated dilation of the brachial artery (at baseline and after 6 months of optimized therapy to improve risk factors) as a measure of endothelial dysfunction in patients with newly diagnosed stable coronary heart disease and reported that persistent impairment of the flow-mediated dilation of the brachial artery is an independent predictor of cardiovascular events (hazard ratio 2.9, 95% confidence interval 1.5–6.2) [51]. Conversely, endothelial function can also identify patients with favorable responses to lifestyle changes such as exercise, weight reduction and smoking cessation, as well as to pharmacological interventions [50].

**Figure 3:**
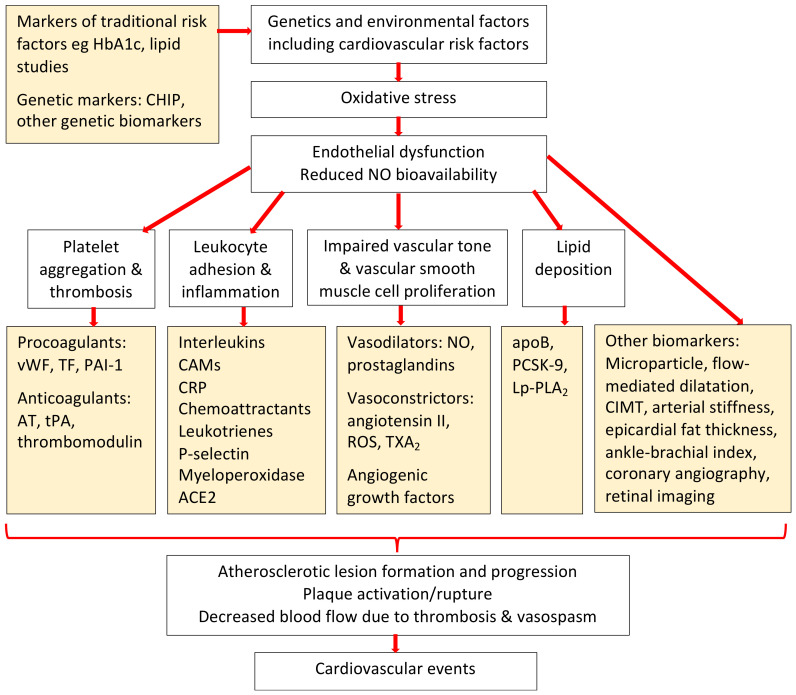
A simplified diagram of some of the endothelial biomarkers available to measure endothelial function (Abbreviations: HbA1c glycated hemoglobin; CHIP clonal hematopoiesis of indeterminate potential; NO nitric oxide; vWF von Willebrand factor; TF tissue factor, PAI-1 plasminogen-activator-inhibitor-1; AT antithrombin; tPA tissue plasminogen activator; CAMs cellular adhesion molecules; CRP C-reactive protein; ACE2 angiotensin converting enzyme 2; ROS reactive oxygen species; TXA_2_ thromboxane A2; apoB apolipoprotein B; PCSK-9 proprotein convertase subtilisin/kexin type 9; Lp-PLA_2_ lipoprotein-associated phospholipase A2; CIMT carotid intima media thickness).

Another method of assessing endothelial function is through the use of biomarkers including those that are lipid-related, inflammation-based or components of renin-angiotensin system. Some of the lipid-related biomarkers include PCSK9 (protein convertase subtilisin/kexin type 9), which promotes the degradation of hepatocyte LDL receptors [52]; apolipoprotein B, found in atherogenic lipoproteins [53]; and the pro-inflammatory Lp-PLA_2_ (lipoprotein-associated phospholipase A_2_), which depletes oxidized phospholipids from lipoproteins [54]. All three biomarkers have shown potential to predict incident cardiovascular disease [52,53,54]. Figure 3 highlights some of the available endothelial biomarkers.

The inflammatory markers include myeloperoxidase, C-reactive protein (CRP), interleukin-6 and GDF-15 (growth-differentiation factor 15). Elevated myeloperoxidase level, released during neutrophil activation, has been associated with coronary artery disease [55], while high-sensitivity CRP has been shown to be associated with cardiovascular events and deaths, incident diabetes and hypertension in several epidemiologic cohort studies [56]. The Women’s Health study suggested that the addition of high-sensitivity CRP to standard Framingham risk factors improved the estimation of future risks of cardiovascular disease [57]. The PLATO trial of over 16,000 participants, on the other hand, found that elevated GDF-15 was associated with increased cardiovascular events and deaths [58].

Another emerging mechanism for the development of cardiovascular disease is via the activation of the renin–angiotensin system. Within this system, angiotensin I is converted to the vasoconstrictor and pro-atherosclerotic angiotensin II by angiotensin converting enzyme (ACE). Angiotensin-converting enzyme 2 (ACE2) is an enzyme that acts to counterbalance the renin–angiotensin system by degrading angiotensin II [59,60]. ACE2 is highly expressed in the endothelium of blood vessels, and it can be shed into the circulation [61]. Although circulating ACE2 activity is low in healthy individuals, it is increased in individuals with cardiovascular risk factors and/or diseases [62,63] such as hypertension, heart failure [64], type 1 diabetes [65] and chronic kidney disease [66,67,68]. In addition, increased circulating ACE2 activity is associated with adverse long-term cardiovascular outcomes [69,70,71].

### 4.3. Vessel Flow

The third contributing factor in Virchow’s triad is flow stasis. Atherosclerosis is a chronic disease that manifests its clinical phenotype through luminal narrowing or by thrombi that obstruct the blood flow to critical organs such as the heart and brain. Sites with low or oscillatory endothelial shear stress are most susceptible [72]. The early lesions consist of subendothelial accumulations of foam cells or cholesterol-laden macrophages, which are the precursors of more advanced lesions. Plaque rupture is the most common culprit resulting in life-threatening thrombosis, as the extremely thin fibrous cap ruptures and exposes the highly thrombogenic, red-cell-rich necrotic core to the blood. Another common mechanism is plaque erosion, in which thrombi form on lesions such as pathological intimal thickening and fibroatheromas without rupture. The clinical manifestations of the thrombotic response in plaque ruptures or erosions are heterogeneous and are likely determined by the combination of the elements of the classic triad of Virchow (the thrombogenicity of the exposed plaque material, local flow disturbances and systemic thrombotic propensity) [72].

Imaging of vessel flow to measure the burden of atherosclerosis may provide better risk assessment than risk assessment calculators. Coronary artery calcium (CAC) scoring can be helpful for determining the extent of coronary artery plaque, while coronary computed tomography angiography (CCTA) provides very high sensitivity for the detection of coronary artery stenosis and nonobstructive plaques. CAC is used in risk assessment in asymptomatic individuals, while CCTA is more commonly performed in symptomatic patients within the lower range of the clinical likelihood of coronary artery disease [73,74]. The PROMISE trial, which randomized over 10,000 symptomatic patients to testing with CCTA or functional testing, found the strategy of initial CCTA to be comparable with functional testing [75]. A CAC score of zero or the absence of stenosis in CCTA are both associated with good prognosis. The CONFIRM trial evaluated the use of CCTA in over 24,000 patients without known coronary artery disease and found that while nonobstructive and obstructive coronary artery disease by CCTA are associated with higher rates of mortality, the absence of coronary artery disease is associated with a low rate of incident death [76].

Retinal vascular imaging is a relatively new non-invasive technology for assessing cardiovascular disease risk [77,78]. The changes in retinal microvasculature have been associated with early-life cardiovascular risk factors including lower birth weight, lesser physical activity, parental hypertension history, childhood obesity and type 1 diabetes [78]. Using a dataset of more than 70,000 images, Cheung et al. recently reported that central retinal vessel caliber measurements correlated well with cardiovascular risk factors including blood pressure, body mass index, total cholesterol and glycated hemoglobin (HbA1c) levels. The study also reported that baseline measurements of the retinal vessel caliber were associated with incident cardiovascular disease [77]. A potential alternative to retinal vascular imaging is the measurement of carotid–intima media thickness (CIMT). Carotid ultrasound is safe and non-invasive and rapid progressive CIMT has been correlated to more cardiovascular and stroke events [79].

## 5. Genetic Insights into Cardiovascular Risk and Disease

The incidence of cardiovascular disease increases with age. While traditional risk factors account for a large proportion of cardiovascular disease, many persons with atherosclerosis do not have established risk factors, and residual risk exists in patients despite optimal medical therapy, suggesting the presence of unknown risk factors. The majority of cardiovascular risk factors and disease are polygenic, with contributions from both heritable and environmental contributions [80,81]. Genome-wide association studies (GWAS) have led to the discovery of hundreds of loci associated with cardiovascular diseases in the last 15–20 years and have further extended our understanding and provided additional insights into the underlying biological pathways, as well as provide a new potential tool for risk calculation, prediction of treatment efficacy or detection of individuals prone to drug side effects [81]. For instance, the CARDIoGRAM study involving more than 22,000 cases and 60,000 controls found an association between a risk variant at 9p21 and a 29% increase in risk of myocardial infarction per copy [82]. Further research to incorporate genomic DNA into risk calculators is underway but will likely be the forefront driver of precision cardiovascular medicine.

An exploratory analysis by Jaiswal et al. reported that carriers of the age-related clonal hematopoiesis of indeterminate potential (CHIP) are at increased risk for not only hematological malignancies and all-cause mortality but also for coronary heart disease (hazard ratio 2.0; 95% confidence interval (CI) 1.2–3.4) and ischemic stroke (hazard ratio 2.6; 95% CI 1.4–4.8) [83]. CHIP, defined as the presence of an expanded somatic blood-cell clone in persons without other hematologic abnormalities, is more common in older persons, with more than 10% of persons over age 70 years carrying this mutation [84]. Jaiswal et al. also reported that participants with CHIP had 4.0 times greater risk of myocardial infarction than non-carriers (95% CI 2.4–6.7) and increased coronary-artery calcification [84]. Moreover, mice engineered to bear the mutations also demonstrated accelerated atherosclerosis. While the mechanisms behind the causal associations between CHIP and cardiovascular disease are still being investigated, it is now known that the most commonly described CHIP mutations in patients with cardiovascular disease are DNMT3A, TET2, ASXI1, TP53, JAK2 and SF3B [85,86]. Some animal models have demonstrated that CHIP amplifies inflammatory responses, while clinical studies have observed that CHIP carriers have increased levels of inflammatory markers [86], supporting the association between aging, CHIP and inflammation.

## 6. Clinical Implications and Future Prospects

The challenges and limitations of our current risk assessment models highlight the need for the further augmentation of our risk prediction paradigm. This model needs to be accurate, reproducible, standardized, inexpensive and easy to interpret with high sensitivity and specificity as well as independent of established predictors [87]. Given the complex interactions within the vascular biology, a single marker is unlikely to be sufficient, which highlights the importance of evaluating the various components of Virchow’s triad in tandem alongside clinical risk factors to develop a comprehensive and personalized cardiovascular risk assessment model. Hence, a multimodality, integrated approach should be adopted. We have recently demonstrated a risk prediction model using a combination of global coagulation assays, age, gender and HbA1c in diabetes patients to be superior to HbA1c alone in predicting diabetic complications [37]. New data from our work and of others demonstrate that biomarkers such as global coagulation assays and endothelial markers may be reporters of the biological effects of traditional risk factors. It is conceivable that scores including these novel biomarkers may add more value to predicting how the combined effects of the traditional risk factors translate into the development of cardiovascular events.

We propose that the next crucial step is to incorporate the assessment of other elements of Virchow’s triad in the development and validation of clinical risk assessment models for cardiovascular and thrombotic diseases. The proposed approach aims to improve the precision of individualized cardiovascular risk prediction through the incorporation of a combination of biomarkers that assess all the components of the triad as well as the clinical surrogate markers. This approach allows for a more complete assessment of individual risks compared with current clinical risk assessment models, which do not take into account patient-specific issues and non-traditional risk factors. Furthermore, this multimodality approach may also be applicable as a secondary cardiovascular risk assessment tool, another deficiency of current models. The ongoing focus of our laboratory is applying the enhanced risk assessment models to patient samples from large-cohort cardiovascular registries and potentially correlate with other future technologies such as GWAS and CHIP. An individualized and precise risk calculator will provide patient-specific risk assessment of future cardiovascular events and enable the early intervention and prevention of cardiovascular disease, an approach that is likely to be cost-effective, reduce burden of disease and save lives.

## 7. Conclusions

Even though cardiovascular diseases account for significant global morbidity, mortality and health expenditure, our current ability to risk stratify individuals is lacking. A more specific and sensitive risk assessment model is required to allow clinicians to confidently recommend early interventions with favorable risk–benefit profiles. Critical to the development of this model is the assessment and understanding of Virchow’s triad and its complex interactions between hypercoagulability, endothelial dysfunction and vessel flow. To achieve this, individualized risk assessment can be improved through an integrated, multimodal approach using a combination of tools such as global coagulation assays, endothelial biomarkers and vessel flow assessment in tandem with clinical surrogate markers. The addition of new tools such as GWAS may further refine the risk stratification.

## Figures and Tables

**Table 1:** A summary of some of the available primary prevention risk calculators.

	Australian Absolute CVD Risk Calculator [21]	ASCVD Risk Estimator Plus [22]	Framingham General CVD Risk Score [23]	SCORE2 [24]	QRISK3 [25]
Year	2012	2013	2008	2021	2018
Components					
Race		√			√
Gender	√	√	√	√	√
Age	√	√	√	√	√
Total cholesterol	√	√	√	√	√
HDL	√	√	√	√	√
LDL		√		√	
Systolic blood pressure	√	√	√	√	√
Diastolic blood pressure		√			
Anti-hypertensives		√	√		√
Diabetes	√	√	√		√
Smoking	√	√	√	√	√
Location				√	√
Others	ECG LVH	Statin Aspirin			Family history, body mass index, chronic kidney disease, SLE, migraine, atypical antipsychotics, corticosteroids, mental illness, erectile dysfunction
Age range (years)	35–74	40–79	>30	40–69	25–84
Risk projection	5-year risk	10-year risk	10-year risk	10-year risk	10-year risk
Endpoints assessed	MI Stroke	Nonfatal MI CHD death Fatal/nonfatal stroke	CHD death Nonfatal MI Angina Fatal/nonfatal stroke Intermittent claudication Heart failure	CHD death Nonfatal MI AnginaFatal/nonfatal stroke Intermittent claudication Coronary revascularization	CHD death Nonfatal MI AnginaFatal/nonfatal stroke Intermittent claudication Coronary revascularization
Webpage	https://www.cvdcheck.org.au/calculator (accessed on 1 June 2022)	https://tools.acc.org/ASCVD-Risk-Estimator-Plus/ (accessed on 1 June 2022)	https://www.ahajournals.org/doi/10.1161/circulationaha.107.699579 (accessed on 1 June 2022)	https://www.heartscore.org/en_GB (accessed on 1 June 2022)	https://qrisk.org/three/ (accessed on 1 June 2022)

AHA American College of Cardiology/American Heart Association; CHD coronary heart disease; CVD cardiovascular disease; ECG electrocardiography; LVH left ventricular hypertrophy; MI myocardial infarction; SLE systemic lupus erythematous; TIA transient ischemic attack.

## Data Availability

Not applicable.
